# Assignment of isochores for all completely sequenced vertebrate genomes using a consensus

**DOI:** 10.1186/gb-2008-9-6-r104

**Published:** 2008-06-30

**Authors:** Thorsten Schmidt, Dmitrij Frishman

**Affiliations:** 1 Department of Genome-Oriented Bioinformatics, Wissenschaftszentrum Weihenstephan, Technische Universität München, D-85350 Freising, Germany; 2 Institute for Bioinformatics and Systems Biology (MIPS), Helmholtz Zentrum München - German Research Center for Environmental Health (GmbH), Ingolstädter Landstraße, D-85764 Neuherberg, Germany

## Abstract

We show that although the currently available isochore mapping methods agree on the isochore classification of about two-thirds of the human DNA, they produce significantly different results with regard to the location of isochore boundaries and isochore length distribution. We present a new consensus isochore assignment method based on majority voting and provide IsoBase, a comprehensive on-line database of isochore maps for all completely sequenced vertebrate genomes.

## Abstract 2

A new consensus isochore assignment method and a database of isochore maps for all completely sequenced vertebrate genomes are presented.

## Background

More than three decades ago gradient density analyses of fragmented DNA identified long compositionally homogenous regions on mammalian chromosomes, widely known as isochores [1-3] or long homogeneous genome regions [4], associated with a wide range of important biological properties. Gene density is up to 16 times higher in GC-rich isochores than in GC-poor isochores [5] (with GC referring to the percentage of the nucleotides guanine and cytosine), and the genes in the GC-rich isochores code for shorter proteins and are more compact with a smaller amount of introns [6]. It was also shown that the GC-rich codons, such as those coding for alanine and arginine, are more frequent in GC-rich isochores [7,8]. The distribution of repeat elements is influenced by the isochore structure of the genome: SINE (short-interspersed nuclear element) sequences tend to be more frequent in GC-rich isochores while the LINE (long-interspersed nuclear elements) sequences are preferentially found in GC-poorer regions [9-11]. The structure of chromosome bands also correlates with isochores: T-bands predominantly consist of GC-rich isochores, while the GC-poorer isochores are found in G-bands [12-14]. The recombination frequency is higher [15,16] and replication starts up to two hours earlier [17] in regions with high GC content.

Further progress in understanding the biological role and evolution of long-range variation in base composition is seriously hindered by the lack of objective and generally accepted isochore assignment methods. A multitude of computational approaches has been developed by various groups [18-23], but no single resource allows the accession, comparison, and combination of isochore assignments made by various techniques in different genomes. Here we introduce a new consensus predictor that characterizes the level of support for isochore locations determined by individual methods. We present a database of isochore maps for all completely sequenced vertebrate genomes and interactive viewers that allow the exploration of this "fundamental level of genome organization" [24] online [25].

## Results and discussion

### Computational methods differ significantly in terms of assigned isochore borders and length

**Figure 1:**
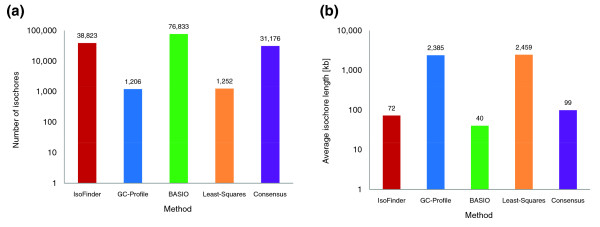
Comparison of isochore assignments in the human genome made by the different methods. All isochore maps show remarkable differences with respect to the number and the average length of their isochore segments. The IsoFinder and BASIO methods result in the most fine-grained segmentations while GC-Profile and least-squares produce less fragmented partitioning of the genome. The consensus map provides a compromise solution. **(a) **Number of isochore stretches. **(b) **Average isochore length.

Published isochore datasets show remarkable diversity. In the following we will use the human genome for comparisons of different isochore assignments if not stated otherwise. The number of isochore segments found in the human genome ranges from about 1,200 for GC-Profile to up to more than 76,000 for BASIO. As a consequence, the resulting isochores show very different length distributions. Isochores discovered by least-squares segmentation are the longest at an average of 2,459 kb, whereas BASIO and IsoFinder segments are the shortest at an average of 40 and 72 kb, respectively (Figure 1). It can be seen that IsoFinder and BASIO are clearly in a different league compared to GC-Profile and least-squares in terms of the number and average length of isochores. This divergence results from different criteria used by the four tested methods to determine the beginning and end of the segments, and the window lengths of 10 and 100 kb used by BASIO and least-squares, respectively. As explained in Materials and methods, a difficult challenge in GC-content-based partitioning of complex eukaryotic genomes is to find a set of parameters suitable for coping with the significantly different levels of GC fluctuations in the GC-rich and GC-poor regions.

**Figure 2:**
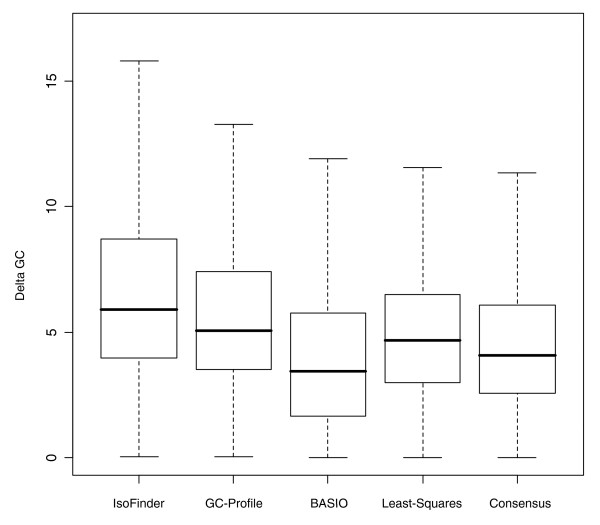
GC differences between neighboring isochores. The distribution of GC differences between adjacent isochores is shown for each method. The thick bars within each box plot indicate the median. The IsoFinder and GC-Profile assignments have the largest GC deltas, on average, whereas in the BASIO isochore map the GC deltas are lowest (median 3.5, mean 4.0). Outliers are not shown in this plot. The average delta GC in the consensus map is 4.6, the median 4.1.

Using the GC level of each isochore, we evaluated the GC difference (delta GC) between adjacent segments and found that the delta GC distributions of the compared methods are significantly different. The BASIO and the least-squares data show the smallest GC jumps while the GC-Profile and IsoFinder methods produce the broadest distribution and the greatest delta GC values on average (Figure 2). One explanation for this may be that short isochores are more likely to model local GC outliers, which results in higher delta GC differences between adjacent segments, on average.

We further assessed the differences between the segmentation methods based on the entropy distance between them. Lower entropy distance values indicate a better agreement between two isochore maps. As shown in Table 1, the results of the least-squares and BASIO approaches are the most dissimilar as measured by this criterion. It is noteworthy that the positions of about 25% of the borders of the least-squares map are identical to those of the BASIO segmentation. This exact border coincidence is an exception, however; in most of the cases segment borders are shifted by between 10 kb and 100 kb for the methods. No borders are shifted by more than 1 Mb with regard to the BASIO borders (Additional data file 1).

**Table 1:** Entropy distance

	IsoFinder	GC-Profile	BASIO	Least-squares	Consensus	Average*
IsoFinder	0.00	1.28	0.53	1.26	0.28	1.02
GC-Profile	1.28	0.00	1.57	0.25	1.20	1.03
BASIO	0.53	1.57	0.00	1.61	0.44	1.23
Least-squares	1.26	0.25	1.61	0.00	1.24	1.04
Consensus	0.28	1.20	0.44	1.24	0.00	**0.79**

Entropy distance was calculated between all segmentations as described in Materials and methods. Higher numbers indicate greater difference between segmentations. The actual classification into particular isochore families is not regarded here. The segmentations of least-squares and GC-Profile are most similar whereas the isochore partitioning of the least-squares and the BASIO method are most distinct. The consensus isochore map is most similar to all other methods on average. *The average agreement of the method in the respective row with all other methods except itself and the consensus isochore map.

### The different methods classify most genomic DNA to the same isochore families

Despite the striking differences between the isochore assignments in terms of segment borders and isochore length, a strong agreement exists with regard to the amount of equally classified DNA and genes. As shown in Table 2, all four original methods assign about 66% of the human genome to the same isochore families. The isochore families are described in detail in the Materials and methods. Furthermore, the four methods locate around two-thirds of all genes in isochores of the same family (Table 3). On average, the consensus in attributing genes to the same isochore between each individual method and the three other methods is between 60.1% (IsoFinder) and 62.4% (least-squares).

**Table 2:** The amount of genomic DNA in which methods agree (%)

	IsoFinder	GC-Profile	BASIO	Least-squares	Consensus	Average*
IsoFinder	100.0	62.2	74.8	58.8	82.3	65.3
GC-Profile	62.2	100.0	59.9	83.1	72.8	68.4
BASIO	74.8	59.9	100.0	60.9	85.5	65.2
Least-squares	58.8	83.1	60.9	100.0	73.7	67.6
Consensus	82.3	72.8	85.5	73.7	100.0	78.6

Percentage of the human genome classified into the same isochore families by each pair of methods. The amount of equally classified human DNA ranges from 59-86% in an all-against-all pairwise comparison. On average, all methods agree in about 66% of the genome. The consensus isochore map has the best agreement of 79%, on average, with all other methods. *The average agreement of the method in the respective row with all other methods except itself and the consensus isochore map.

**Table 3:** Agreement on gene classification (%)

	IsoFinder	GC-Profile	BASIO	Least-squares	Consensus	Average*
IsoFinder	100.0	53.1	76.5	50.7	81.1	60.1
GC-Profile	53.1	100.0	54.6	83.8	68.9	63.8
BASIO	76.5	54.6	100.0	52.6	83.7	61.2
Least-squares	50.7	83.8	52.6	100.0	66.7	62.4
Consensus	81.1	68.9	83.7	66.7	100.0	75.1

Percentage of genes that are classified equally by all methods. Between 50% and 84% of all genes are classified into the same isochore family by all methods. The consensus isochore map shows the greatest agreement with all other isochore maps on average. *The average agreement of the method in the respective row with all other methods except itself and the consensus isochore map.

The breakdown of the genome into the five isochore families is very similar for all the methods. On average, 22 ± 2.5% (standard deviation) of the complete human DNA is found in the L1 isochore. The most dominant isochore family is L2, with 34 ± 2.7% of the DNA, followed by the H1 family with 23 ± 1.5%. The remaining 15% of the genome is distributed between the H2 and H3 families, with 11.4 ± 0.2% and 3 ± 1.1% of the DNA, respectively. The low deviation values among the methods indicate a good overall agreement between all the isochore maps.

### Properties of the human consensus isochore map

**Figure 3:**
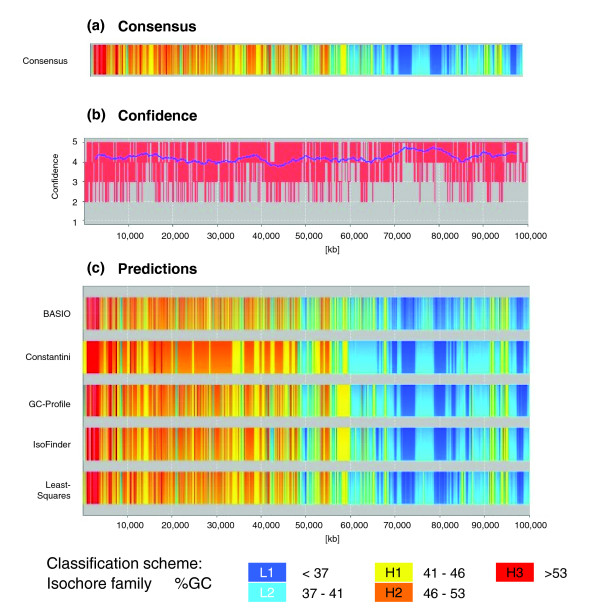
Graphical representation of the isochore assignments for the first 100 Mb of the human chromosome 1 (obtained from the IsoBase web page [25]). **(a) **Consensus assignment. The color code depicts the isochore families as defined by Bernardi *et al*. [26,18]**(b) **Confidence of the assignments. For each residue the number of isochore methods that support a given isochore class is depicted as a red line. Support values for individual bases are averaged over a sliding window (blue line). **(c) **Isochore predictions made by each of the available methods.

Significant similarities between the DNA and gene classifications produced by the different computational methods render a consensus isochore assignment feasible. As outlined in the Materials and methods, the consensus assignment assumes the isochore family that is predicted by the majority of methods at each genomic position. This simple consensus approach results in 31,176 distinct isochores in the human genome, with an average isochore length of 99 kb (Figure 1). The median and average delta GC differences between neighboring isochores are 4.1 and 4.6, respectively (Figure 2). With regard to the number, length and delta GC values of isochores, the consensus assignment shows a reasonable balance between the observed extreme values of the individual methods. The amount of ambiguous DNA, that is, the nucleotides that could not be classified by the majority approach, is less than 0.2%. Our interactive online isochore browser (Figure 3) allows for a visual comparison between the individual isochore assignment methods and the consensus isochore map.

### Evaluation of the fit to biological models

Due to the lack of large-scale experimental data on isochore location in the human genome, we are evaluating whole-genome isochore assignments using indirect evidence by considering independent biological properties known to be associated with GC content variation. One such property is gene density (the number of genes per Mb) which is known, to vary significantly between different isochore families of the human genome [5,26,27], from very high in H3 to very low in L1. This observation was first made experimentally and subsequently confirmed by genome sequencing; for a review of possible causes, see [24,27-29]. A biologically meaningful genome segmentation would thus be expected to display a strong correlation with gene density.

**Figure 4:**
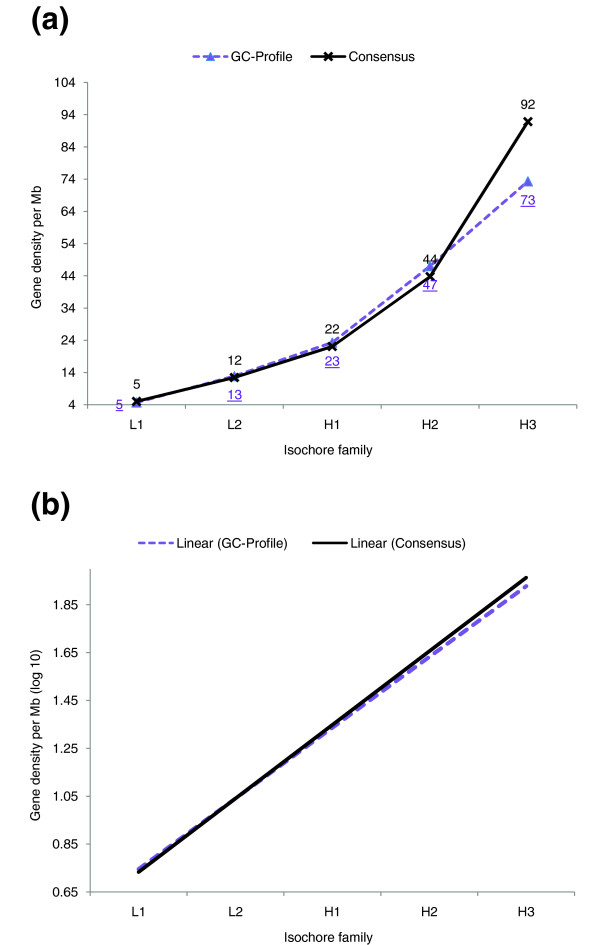
Correlation between isochore classification and gene density. **(a) **A comparison of the gene density in the consensus isochore map and the GC-Profile segmentation. The underlined data labels denote the gene densities of the GC-Profile segmentation, the non-underlined labels the gene densities of the consensus map. In the consensus assignment more genes can be found in the H3 isochore family than in the GC-Profile assignment. The consensus assignment thus provides a stronger signal in terms of the expected correlation between gene density and isochore class. **(b) **Linear regression lines of the logarithmized (base 10) gene density values for the isochore families L1 to H3. The isochore families were numbered from 1 to 5 to compute the regression. The slope of the regression line is slightly greater for the consensus isochore map.

We compared the different isochore maps with respect to the degree of correlation between genome segmentation and gene density. As an example, Figure 4a shows a comparison between GC-Profile and the consensus method. Both methods display a clear dependence on the isochore classification of genomic regions, with gene density varying over a broad range between 5 (for both GC-Profile and the consensus map in the L1 isochore) and 73 or 92 (for GC-Profile and the consensus map, respectively, in the H3 isochore). The consensus assignment thus conforms better to the intuitive isochore-gene density model in that it displays higher gene density in the H3 isochore (Figure 4a). Therefore, the consensus isochore assignment provides a stronger signal in terms of gene density-isochore correlation than the GC-Profile segmentation.

The strength of the correlation between two variables can be estimated in a more rigorous way based on the slope of their respective linear regression lines, as shown in Figure 4b. The greater the slope of the consensus regression line the stronger the association between the resulting segmentation and gene density compared to GC-Profile. As seen in Table 4, the slope of the consensus isochore map is steeper than that of all other methods, signifying that the consensus approach is the most valid one with respect to this particular biological feature.

**Table 4:** Isochores and gene density

Source of gene models	IsoFinder	GC-Profile	BASIO	Least-squares	Average*	Consensus
UCSC known genes	0.696	0.681	0.703	0.693	0.693	**0.708**

For each isochore map, the gene density (number of genes per Mb) in each of the isochore families L1 to H3 was calculated. Shown is the slope of a linear regression line of the logarithmized densities versus the isochore families. For computing the regression, the isochore families were treated as numbers, from 1 for the L1 family to 5 for the H3 family. Firstly, one can see that gene density is positively correlated with isochore families as all values are positive. Secondly, the consensus isochore map explains gene density best as the slope of the consensus method is greatest. A greater line slope means less gene density in the L isochores and a higher gene density in the H isochores. This is exactly what would be expected in a model with the best fit to the biological hypothesis. *The average gene density of all methods except the consensus isochore map.

### Evaluation with regard to experimentally confirmed isochore data

In addition to our genome-wide analysis of gene density, we carefully analyzed currently available direct experimental evidence pertinent to isochore properties (Table 5). For each of the five computational methods (IsoFinder, GC-Profile, BASIO, least-squares, and the consensus approach) we investigated whether or not they meet the respective criteria. The first two tests took advantage of the recent experiments of Schmegner *et al*. [30]. In their work, they showed that the human *MN1 *gene (residing in a GC-rich isochore) is replicated several hours earlier (during the S phase of the cell cycle) than the neighboring gene *PITPNB *from a GC-poor isochore. Furthermore, a second isochore border within the human KIAA1043 gene was described and experimentally verified. As seen in Table 5, the first border between *MN1 *and *PITPNB *was correctly recognized by all methods except for the least-squares approach. The second border in the KIAA1043 gene was not detected by the least-squares or the GC-Profile assignments. We are aware that these failures may be overcome by further tuning of these methods, although this will give rise to a host of new questions. However, all isochore borders are correctly found by the consensus approach. In a further test, we checked the detection of the well known isochore border between the genes encoding the human MHC class II and class III region [17]. This border is correctly found by all methods. This is not surprising as all methods were evaluated against the available body of experimental evidence at the time of their publication and fine-tuned by their respective authors.

**Table 5:** Experimental evaluation

				Method meets criteria				
				
Evaluation criteria		Experimental evidence	References	IsoF	GC-P	BASIO	L-S	Consensus
1.	Isochore border between the genes *MN1 *(in the GC rich region) and *PITPNB *(in the GC poor region) in the human genome	Replication time during the S phase of the cell cycle	[30]	Yes	Yes	Yes	**No**	Yes
		Early *MN1 *gene, late *PITPNB *gene						
		Pause of about 3 hours at isochore border						
2.	Isochore border within the KIAA1043 gene in the human genome	Replication time during the S phase of the cell cycle	[30]	Yes	**No**	Yes	**No**	Yes
		Long pause at isochore border						
3.	Isochore border between the MHC classes II and class III regions	Replication time during the S phase of the cell cycle	[17]	Yes	Yes	Yes	Yes	Yes
		Long pause at isochore border						
4.	Typical isochore length and isochore length distribution subject to isochore GC content	Ultra-centrifugation in combination with fragmentations at different scales. See also theoretical discussions in Constantini *et al*.	[2,3,18]	**No**	**Partly**	**No**	**Partly**	Yes

IsoF, IsoFinder; GC-P, GC-Profile; L-S, least-squares.

Finally, we evaluated the isochore length distributions. Early experiments that applied fragmentations at various scales [2,3] as well as theoretical studies [18] suggest a typical isochore length significantly longer than the average size of 72 and 40 kb predicted by IsoFinder and BASIO, respectively, in the human genome. GC-Profile and least-squares meet these isochore length requirements. However, none of the individual methods - except for the consensus method - results in an isochore map that shows an isochore length distribution similar to that annotated by the Bernardi group for an outdated human genome assembly [18]. As summarized in Table 5, the consensus approach appears to be more robust in that it meets all experimentally verified criteria, while all other methods fail in one or more tests. Furthermore, the quality of the consensus assignments is bound to further improve as more complementary isochore prediction methods are incorporated.

### Confidence of isochore assignments and cross-genome comparison

**Figure 5:**
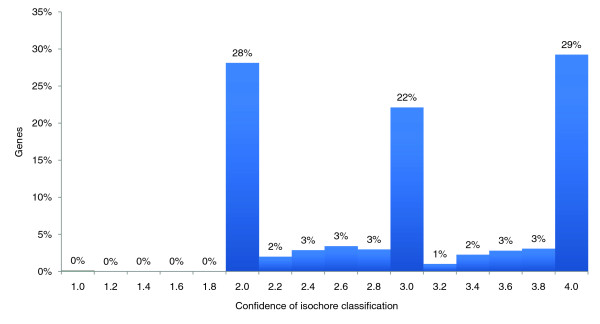
Isochore assignment confidence of human genes. Each bin of the histogram shows the percentage of genes supported by a given average number of computational methods. Denoted is the upper border of each bin. Each bin shows the number of genes having an isochore assignment confidence c with lower-border < c ≤ upper border. For example, 30% of genes have a confidence value of >1.8 and ≤ 2.0. About one-third (29%, the right-most bar) of all genes are equally classified by all four independent methods (BASIO, IsoFinder, GC-Profile and least-squares). Gene classifications with low confidence can hardly be found. For 99.8% of all genes at least two methods agree completely over the whole coding region. Furthermore, only very few genes have a confidence value between two full numbers. This can be explained by two observations: the genes are usually completely located within a single isochore stretch; and these gene regions are hardly separated by any of the segmentation methods. Therefore, usually two, three or all four methods agree for the complete gene. The mean and median support for all genes is 3.0.

Most genes completely reside within a single isochore stretch (Additional data file 2). A comparison of random segmentations that have comparable block lengths shows that more genes are wholly located within an isochore segment than would be expected by chance. This is especially pronounced in isochore segmentations with segments of relatively short average length, such as those determined using IsoFinder and BASIO, and underlines the utility of isochore information for gene prediction. This observation may be related to the structure of chromatin [31] or chromosome break-prone regions [32]. We also found that most genes are classified into the same isochore families by the different methods. As a consequence, the isochore assignment confidence, as defined in Materials and methods, is very good for most genes and hardly any genes are classified with low confidence (Figure 5). One further observation is that most genes are found in regions with integer confidence values. This can be explained by the fact that genes typically reside completely within a single isochore stretch, irrespective of the applied method. For example, if a gene is completely covered by an isochore stretch in all isochore predictions, then the confidence value for this gene will always be two, three or four, depending on the number of methods that agree in their classification. In contrast, non-integer confidence values indicate regions that show a certain agreement for parts of the gene only, usually because an isochore border is located within a given gene. Overall, 99.8% of all genes are assigned to the same isochore families by at least two methods. This provides a sound basis for using isochore classification of genes in experimental studies such as expression analysis.

**Figure 6:**
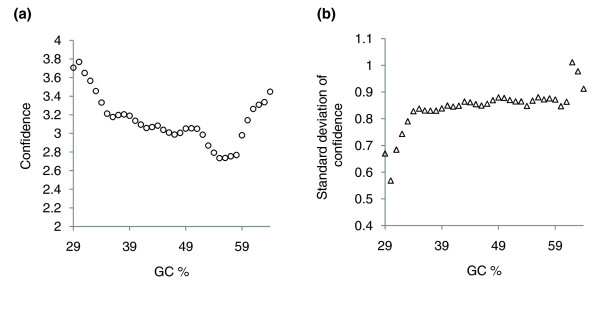
Isochore assignment confidence and GC context. **(a) **Confidence as a function of the GC content of the genomic environment. Isochore assignment confidence is best in GC-poor regions; it decreases as the genomic context becomes more GC-rich and reaches a minimum around 55-58% GC. However, the assignment confidence becomes better again in the GC-richest regions with >59% GC. **(b) **Variance of the confidence depending on the GC content. The confidence variance is independent of the GC context for isochores with a GC content between about 33% and 62% GC, that is, for the main bulk of the genomic DNA.

**Figure 7:**
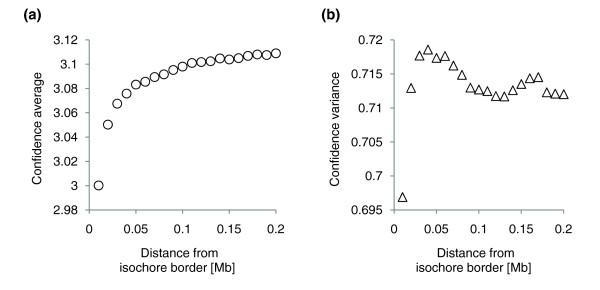
Isochore assignment confidence in border regions. **(a) **On average the isochore assignment confidence is lowest near borders. Here the borders of all isochore maps were used. Assignment confidence grows with the distance from the border and reaches saturation at a distance of about 0.2 Mb from the border. This can be considered as an empirically derived 'safe distance' threshold. **(b) **Variance of the assignment confidence is almost independent of the border distance.

Overall, the confidence of the isochore assignment in the human genome is higher in GC-poor regions (Figure 6). The confidence decreases in GC-richer regions and reaches a minimum at GC content values around 55-58%. This may be explained by the increasing GC fluctuations in GC-richer regions [33]. Elevated confidence levels corresponding to the lowest and highest GC levels may be explained by simple statistical reasons. For example, the GC-richest regions are most likely to be classified into one out of two isochore families: the GC-richest H3 family or the less GC-rich H2 family. By contrast, a segment with an intermediate GC content may fall into one of three isochore families (for example, H2, H1 or L1). Given this limited event space, the likelihood of observing an agreement between the methods for the GC-richest and GC-poorest regions will be higher. The isochore confidence is least near isochore borders in all isochore maps (Figure 7). It quickly grows with distance from the borders and reaches saturation at a distance of approximately 0.2 Mb from the border. This empirical observation can be useful for defining a 'safe distance' threshold in practical applications of isochore information, allowing the estimation of the isochore classification reliability at any region of interest even if no consensus or confidence information is to hand.

We calculated isochore assignments and evaluated their confidence for 20 completely sequenced vertebrate genomes using GC-Profile, IsoFinder, least-squares and BASIO as well as our consensus method (Tables 6 and 7). The amount of DNA that could not be classified by majority vote into one of the five isochore families in our consensus maps for any of these 20 genomes was very small, less than 1% on average. The overall isochore assignment confidence is generally very high, with 2.6 methods agreeing on average. The entropy distance between the consensus map and the segmentations of all four individual methods indicates to which isochore segmentation the consensus map is most similar. This large-scale comparison shows that there is neither a single method clearly closest to the consensus, nor a simple dependency of a method's performance on the overall GC-richness of the genomes.

**Table 6:** Database content

					Entropy distance: consensus and each method				
						
Genome		Version	Source*	Size^†^	IsoF	GC-P	BASIO	L-S	GC %
*Bos taurus*	Cow	bostau4-0	HGSC	2.5	0.3	1.9	0.3	2.0	41.8
*Canis familiaris*	Dog	canfam2	UCSC	2.5	0.3	1.3	0.3	1.2	41.3
*Danio rerio*	Zebrafish	danrer4	UCSC	1.5	0.4	2.1	0.4	2.3	36.5
*Danio rerio*	Zebrafish	danrer7	Ensembl	1.3	0.3	1.9	0.5	1.9	36.5
*Drosophila melanogaster*	Fruit fly	dm2	UCSC	0.13	0.4	1.7	0.4	1.8	42.2
*Equus ferus caballus*	Horse	equCab1	UCSC	2.0	0.3	1.4	0.3	1.4	41.5
*Gallus gallus*	Chicken	galgal3	UCSC	1.0	0.6	0.4	0.6	0.5	41.3
*Gasterosteus aculeatus*	Stickleback	gasAcu1	UCSC	0.45	0.3	1.9	0.3	1.9	44.6
*Homo sapiens*	Human	hg17	UCSC	3.0	0.3	0.3	0.6	0.3	40.9
*Homo sapiens*	Human	hg18	UCSC	3.0	0.3	1.2	0.4	1.2	40.9
*Mus musculus*	Mouse	mm8	UCSC	2.6	0.3	1.0	0.6	1.1	41.8
*Mus musculus*	Mouse	mm9	UCSC	2.6	0.3	1.1	0.5	1.2	41.8
*Monodelphis domestica*	Opossum	monDom4	UCSC	3.4	1.0	1.4	0.9	1.4	37.8
*Monodelphis domestica*	Opossum	monDom5	Broad	3.4	1.1	1.4	0.9	1.4	37.8
*Ornithorhynchus anatinus*	Platypus	ornAna1	UCSC	0.43	0.4	1.7	0.3	1.8	45.1
*Oryzias latipes*	Medeka	oryLat1	UCSC	0.58	0.4	2.1	0.3	2.2	40.5
*Pan troglodytes*	Chimpanzee	pantro2	UCSC	3.1	0.2	1.6	0.3	1.7	40.7
*Macaca mulatta*	Macaque	rheMac2	UCSC	2.7	0.3	1.8	0.3	2.1	40.6
*Rattus norvegicus*	Rat	rn4	UCSC	2.7	0.3	1.9	0.3	2.1	41.9
*Tetraodon nigroviridis*	Pufferfish	tetNig1	UCSC	0.2	0.3	1.7	0.3	1.7	45.8

*Genome downloaded from: UCSC, University of California Santa Cruz Genome Browser; HGSC, Human Genome Sequencing Center at Baylor College of Medicine; Broad, The Broad Institute; Ensembl, Ensembl genome database. ^†^Genome size in gigabytes, without unassembled parts. IsoF, IsoFinder; GC-P, GC-Profile; L-S, least-squares.

**Table 7:** Database content

			%DNA classified to isochore families in the consensus map						Confidence^‡^	
				
Genome		No. of segments*	L1	L2	H1	H2	H3	Am^†^	Avg.	SD
*Bos taurus*	Cow	48	8.6	45.1	28.4	11.9	3.44	0.14	2.6	0.70
*Canis familiaris*	Dog	36	25.8	32.5	20.3	11.3	5.05	0.16	2.6	0.71
*Danio rerio*	Zebrafish	22	76.6	20.4	1.9	0.4	0.12	0.23	2.6	0.74
*Danio rerio*	Zebrafish	22	78.1	20.1	1.4	0.3	0.08	0.04	2.6	0.76
*Drosophila melanogaster*	Fruit fly	1	2.6	20.5	70.8	5.7	0.06	0.02	2.6	0.76
*Equus ferus caballus*	Horse	32	22.1	37.7	22.8	12.5	4.57	0.16	2.6	0.71
*Gallus gallus*	Chicken	11	16.2	40.3	26.4	10.6	3.23	1.16	2.2	0.58
*Gasterosteus aculeatus*	Stickleback	6	0.0	3.4	73.7	22.2	0.22	0.04	2.7	0.77
*Homo sapiens*	Human	24	22.6	33.6	22.2	11.1	3.20	0.13	2.7	0.73
*Homo sapiens*	Human	31	22.8	33.2	22.7	11.2	3.01	0.13	2.5	0.70
*Mus musculus*	Mouse	15	7.4	40.0	34.9	14.2	0.33	0.02	2.6	0.68
*Mus musculus*	Mouse	17	7.9	39.5	34.6	14.2	0.50	0.01	2.6	0.72
*Monodelphis domestica*	Opossum	32	49.1	36.6	9.7	2.3	0.57	0.54	2.5	0.72
*Monodelphis domestica*	Opossum	32	49.5	35.8	10.2	2.3	0.55	0.64	2.5	0.72
*Ornithorhynchus anatinus*	Platypus	8	0.2	22.4	61.7	14.3	1.25	0.06	2.7	0.75
*Oryzias latipes*	Medeka	16	3.5	55.3	30.7	2.5	0.14	0.12	2.7	0.75
*Pan troglodytes*	Chimpanzee	67	22.4	32.2	21.6	10.5	2.90	0.22	2.6	0.72
*Macaca mulatta*	Macaque	72	24.4	35.2	22.7	11.0	3.06	0.35	2.6	0.71
*Rattus norvegicus*	Rat	63	7.6	38.0	34.5	15.7	0.74	0.12	2.6	0.72
*Tetraodon nigroviridis*	Pufferfish	4	0.1	5.7	49.3	34.8	3.16	0.15	2.6	0.75

*Number of segments in the consensus map in thousands. ^†^Ambiguous amount of DNA that could not be assigned to one of the five isochore families L1 to H3 in the consensus map. ^‡^Assignment confidence of all stretches in the consensus map: average and standard deviation. Avg., average; SD, standard deviation.

We furthermore present in Table 7 the amount of DNA that is found in each of the isochore families for all genomes. As expected, the overall GC content of a genome influences the amount of DNA in the different isochore families in that the genomes that have, on average, higher GC content are supposed to have more DNA in GC-richer isochores. However, a simple correlation could not be found. For example, in the dog genome, 5% of the DNA is in H3 isochores, whereas in the platypus genome only 1% is in the H3 isochores. The opposite would have been expected as the platypus genome has a high overall GC content (46%) in comparison to the much lower GC content (41%) of the dog genome.

### Availability and database content

We have created an online database, IsoBase, where all data described in this study are freely accessible. Our website enables the user to evaluate statistical distributions of isochore properties, and compare isochore assignments within and between organisms and methods. Multiple qualitative and quantitative properties of isochore maps can be interactively explored. Confidence values of each segment are displayed for each consensus isochore map. Tables 6 and 7 show an overview of genomes included in our database and their isochore properties.

For convenience, we provide two search interfaces at our IsoBase website [25]. The first search feature allows the genomic positions and the isochore families of genes to be looked up by free text searches and by multiple identifier types. Currently, genes can be looked up by RefSeq identifiers, UniProt/SwissProt accessions, Ensembl IDs, gene and protein names, as well as by their descriptions, and SwissProt keywords. The second search option allows retrieval of available isochore information for a list of genomic positions in one step. All isochore assignments and the corresponding confidence information can be visualized online and downloaded as tab-delimited data files. In addition, we provide UCSC custom annotation tracks of the consensus isochore assignments for all genomes. All UCSC tracks can be downloaded from our web site. Furthermore, the isochore tracks are integrated into the UCSC view automatically by using the links to the UCSC genome browser at our web site [25].

## Conclusion

We have demonstrated that available isochore assignment approaches produce significantly different segmentations in terms of the location of isochore borders and the GC differences between neighboring stretches. At the same time, the total amount of genomic DNA classified into the same isochore families is very large, with all methods being in perfect agreement for more than two-thirds of the human genome.

The consensus isochore assignment method based on the majority vote at each genomic position has four distinct advantages. First, it provides a more balanced isochore assignment that is more robust against under- and over-fragmentation. Second, it appears to produce more biologically relevant results as judged by better correlation between the resulting segmentation and gene density. Third, evaluation based on experimentally derived isochore data shows that our consensus approach is in better accordance with all the criteria than the individual methods. Finally, our procedure allows the reliability of the isochore assignments to be estimated. We suggest that the consensus method has the potential to be further improved in the future by adding more complementary datasets.

We have demonstrated that most genes reside within a single isochore stretch and can be classified with high confidence. The isochore assignments become very reliable at a distance of about 0.2 Mb from isochore borders. This empirical observation allows the assignment of confidence to be estimated even in the absence of any further knowledge.

In conclusion, we recommend using consensus assignments for best confidence and best accordance with biological models that were found to be associated with isochores. We further demonstrate that the consensus approach is more robust than relying on a single method alone. At our website, IsoBase [25], we provide isochore consensus assignments for all completely sequenced vertebrate genomes along with confidence information for visual exploration, searching and downloading. We will add isochore consensus maps for new genomes as they become available. We hope that this resource will stimulate further analysis and exploration of the large-scale variation of genome properties.

## Materials and methods

### Isochore assignments

We refer to the isochore nomenclature as it was first described based on ultra-centrifugation experiments [26]. Bernardi and colleagues [18] defined the isochores according to their GC content. There are three isochore types with high GC content, H3 (>53%), H2 (46-53%), and H1 (41-46%), and two types with low GC content, L1 (<37%) and L2 (37-41%). In Additional data file 3 we present an analysis of the amount of genomic DNA versus segments' GC content (by 1% bins) and confirm that distinct isochore families can be observed throughout the genomes analyzed in this study. The Bernardi group [18] calculated the GC content of 100 kb long, non-overlapping sequence windows and then merged the windows if the difference in their GC content was below 1-2%. However, no hard threshold was used, and in many cases subjective decisions were made as to whether or not to merge windows, making the Constantini method as described in the original publication hardly fully automatable. In particular, this circumstance makes it impossible to consider the Constantini data for our comparison of isochore assignment methods, which is based on a more recent version of the human genome than the one used in the original publication.

In this work isochores were predicted by four methods for automatic genome segmentation: GC-Profile [22,23], BASIO [21], IsoFinder [20], and least-squares optimal segmentation [19,34]. Briefly, GC-Profile is a windowless method that recursively partitions the input sequence into two subsequences, left and right, based on the quadratic divergence between statistical measures (such as genome order indices, a^2^+c^2^+g^2^+t^2^, where a, c, g, and t are occurrences of individual bases) reflecting base composition. IsoFinder moves a sliding pointer along the input DNA sequence and finds a position that maximizes the GC difference between its left and right portions according to t-Student statistics. Then both portions are split into non-overlapping 300 kb windows, and for each individual window the GC content is computed. If the mean values of the window GC content on the left and the right of the pointer position are significantly different, this position becomes the cutting point and the input sequence is divided into two subsequences. Both GC-Profile and IsoFinder proceed from left to right and may produce different results if the direction is inverted. BASIO calculates Bayesian marginal likelihood for sequence segments and, for reasonably short DNA contigs, attempts to find a global maximum of the overall likelihood over all possible configurations of segment borders using a Viterbi-like dynamic programming algorithm. For large DNA sequences, such as complete chromosomes, BASIO relies on an approximate split-and-merge procedure to find an optimal segmentation. We applied the BASIO method using the default border insertion penalty 3 and 10 kb sequence blocks as initial input. Finally, the least-squares method calculates GC content (values logarithmized as in [19]) in non-overlapping 100 kb windows (default window size as in [19]) and then derives optimal segmentation of the resulting array of real values, which yields the minimal sum of squares of the Euclidian distance between each value and its segment average. However, the least-squares algorithm requires the user to provide the expected number of output segments as a parameter. As an estimate of this number for the least-squares method we utilized the minimum number of isochores produced by the three other methods - GC-Profile, BASIO, and IsoFinder. This approach makes over-fragmentation unlikely and provides a lower limit for the actual number of isochores. All methods are clearly distinct in terms of their methodology; a review of fundamental statistics in segmentation approaches is given in [35]. Additionally we show in Additional data file 3 that all methods make a complementary contribution to the consensus maps throughout all genomes.

Methods that rely on any information beyond the raw nucleotide sequence for isochore prediction were not considered in this study. For example, the Markovian approach of Melodelima *et al*. [36] incorporates information about known biological features such as genes and their properties to create hidden Markov models. By contrast, all the methods in this study are solely based on the GC content and, therefore, can be used even in the absence of reliable gene models, for example, in a newly sequenced genome.

### Genomic data

We used the human genome as a test case for comparing isochore assignments made by the different methods. The latest human genome assembly hg18 (build 36) was obtained from the UCSC genome browser [37]. Further vertebrate genomes were downloaded from UCSC, Ensembl [38], and the Broad Institute [39,40]. Assembly parts marked as random and short scaffold parts were not considered. The 'UCSC known genes' models [41] were used for computing gene density, defined as the number of genes per million nucleotides (Mb). To determine the gene density in individual isochores, we counted the number of genes that start in each isochore family and divided it by the total amount of genomic DNA classified into the respective isochore family. For the regression analysis the isochore family labels were translated into their ordinal value: from 1 for the L1 family to 5 for the H3 family. Gene density values were logarithmized (natural logarithm) as they grow polynominally with increasing isochore family number. Statistical tests were performed using PROMPT [42].

### Entropy distance

In this study we are measuring the distance between two segmentations P and Q by the 'entropy distance' as introduced and discussed by Mielikäinen *et al*. [43] and Haiminen *et al*. [44], respectively. Briefly, the entropy H of a segmentation P with k segments can be defined as:

$$\begin{array}{ccc} H(P)=-\sum_{i=1}^{k}\mathrm{Pr}(p_{i})\log\mathrm{Pr}(p_{i}) & \text{with} & \mathrm{Pr}(p_{i})=\frac{length\;of\;segment\;i}{total\;length\;of\;the\;segmented\;sequence} \end{array}$$

The entropy distance is the conditional entropy of P given Q and *vice versa*. Conditional entropy is thus an information theoretic measure that quantifies the amount of information that one segmentation gives about the other. The lower the entropy distance between the reference isochore segmentation and the prediction, the closer the prediction is to the reference.

As further shown in [44], the conditional probability of the segmentation P given the segmentation Q can be computed with the complexity O(k_p_+k_q_), with k_p _and k_q _being the number of segments in P and Q. This efficient algorithm uses the fact that H(P|Q) = H(U) - H(Q), with H(U) being the entropy of the union of P and Q. Therefore, the entropy distance of P and Q can be represented as:

H(P|Q) + H(Q|P) = 2 H(U) - H(Q) - H(P)

### Consensus isochore assignments

We sought to integrate several available methods in order to provide more balanced isochore assignments. It is known that GC fluctuations tend to be higher in GC-rich regions than in GC-poor regions [33]. This means, for example, that if one partitions human DNA sequence into blocks of 100 kb, the GC content variation between such blocks in a GC-rich region will be higher than in a GC-poor region. A segmentation algorithm that aims at partitioning a genome based on the GC variance must be able to handle these differences. If a method is optimized to detect small GC jumps between genomic blocks, it is likely to overfragment GC-rich regions. Conversely, if the cut-off value of the GC content change required to initiate a new segment is too high, GC changes between different isochores in GC-poor regions will not be detected. The significant variety in the methodology of currently available isochore prediction approaches reflects to some degree this difficult challenge.

Our consensus classifier tackles this issue by integrating all available *ab initio *methods that are fully automatable: IsoFinder, GC-Profile, least-squares and BASIO. For all genomes in our database we provide a consensus isochore map in addition to the assignments calculated by individual methods. Each base position is classified independently by each method into one of the five isochore families - L1, L2, H1, H2 or H3 - as defined by Bernardi *et al*. [26]. The consensus isochore assignment is then made based on the majority vote. Standoff regions are marked as such and classified into the L1 to H3 families by their GC level. For example, a standoff situation can occur if exactly one-half of all methods assign a certain isochore family, for example, L1, whereas the other half of all methods proposes an opposing isochore family, for example, L2. In such a case the decision to choose one isochore family is made based on the GC content level of the affected sequence. Remaining rare positions, where no majority could be found, for example, because all four methods give different results, or where some of the predictions are missing, are marked as ambiguous.

One adjustable parameter of our consensus approach is the genomic resolution at which the majority vote is taken. For those isochore maps based on 0.1 Mb windows (Costantini *et al*. [18], least-squares with default window size [19]) the best resolution would be at the level of 0.1 Mb. Other methods such as IsoFinder [20] determine isochore borders at the level of single bases. Considering that the average isochore length obtained by the four methods used in this study is in the range 0.1-0.9 Mb (see Results), the resolution of 0.01 Mb for deriving consensus is a compromise between these extremes and is used as the default setting in our study. The consensus confidence is defined as the number of methods that agree at a certain genomic position and can thus take values between one and four. The confidence of the isochore assignment for an entire genomic region is computed as the average of all base confidence values.

Random control segmentations were created by partitioning into segments with the average isochore length of the given method, for example, IsoFinder. For the segmentation offset, a random segment length was chosen. For the random model, a normal distribution around the average isochore length was used.

## Authors' contributions

TS conducted the research. DF conceived and directed the work. TS and DF wrote the manuscript. All authors read and approved the final manuscript.

## Additional data files

The following additional data are available with the online version of this paper. Additional data file 1 is a figure showing distances between the isochore borders produced by different methods. Additional data file 2 is a figure showing the percentage of genes that are completely located within a single isochore. Additional data file 3 provides an analysis of isochore families in all genomes and analysis of differences between methods.

## Supplementary Material

Additional data file 1 Most borders are shifted between 10 and 100 kb among all methods. No borders are shifted more than 1 Mb in comparison to the BASIO borders. One exception is the least-squares segmentation, which has identical borders with the BASIO map in about 25% of all cases. Click here for file

Additional data file 2 For all isochore assignments, more genes reside completely within a single stretch than one would expect by chance. All results are statistically significant (Chi-Square test, all *p*-values < 0.001). Click here for file

Additional data file 3 Analysis of isochore families in all genomes and analysis of differences between methods Click here for file
