# Supplementary figures and images for: Assignment of isochores for all completely sequenced vertebrate genomes using a consensus
Source: Genome Biol. 2008 Jun 30;9(6):R104. doi: 10.1186/gb-2008-9-6-r104 (PMC2481423; doi:10.1186/gb-2008-9-6-r104)

IsoFinder GC-Profile Least-Squares

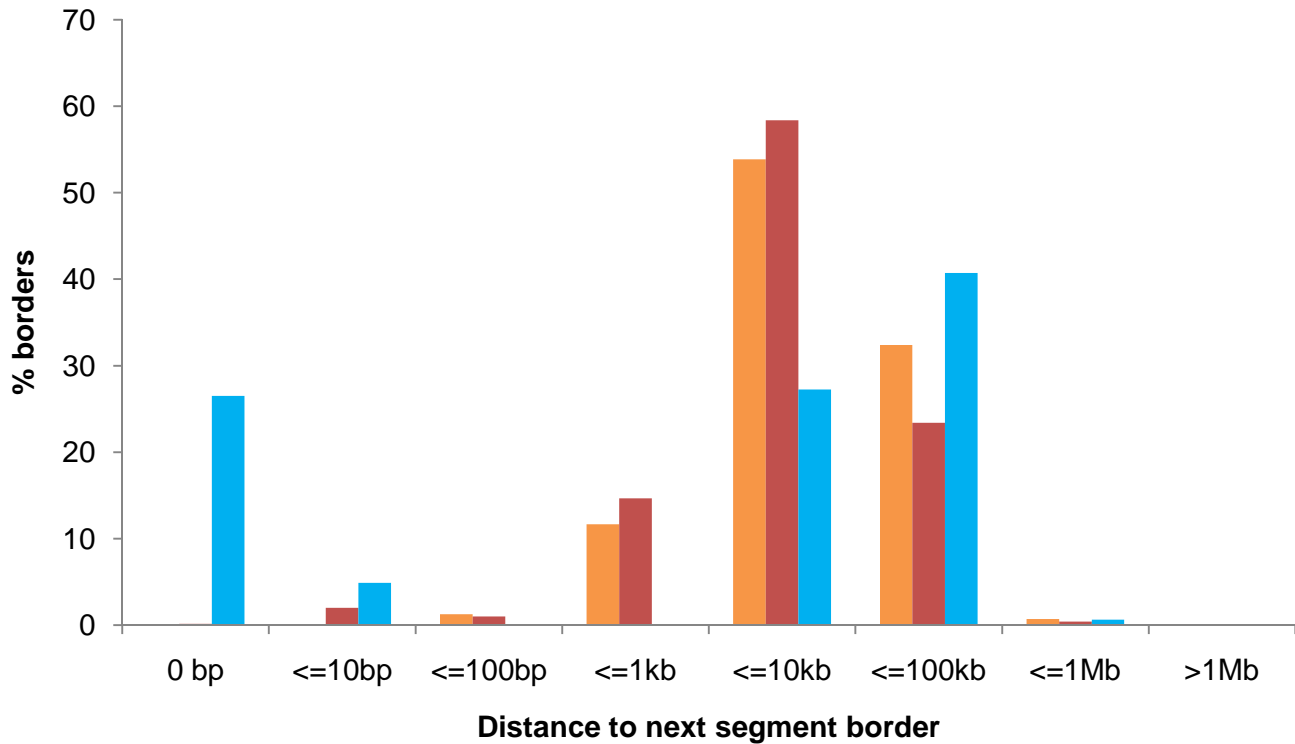

Supplement: Additional data file 1 — Most borders are shifted between 10 and 100 kb among all methods. No borders are shifted more than 1 Mb in comparison to the BASIO borders. One exception is the least-squares segmentation, which has identical borders with the BASIO map in about 25% of all cases. [file gb-2008-9-6-r104-S1.pdf]

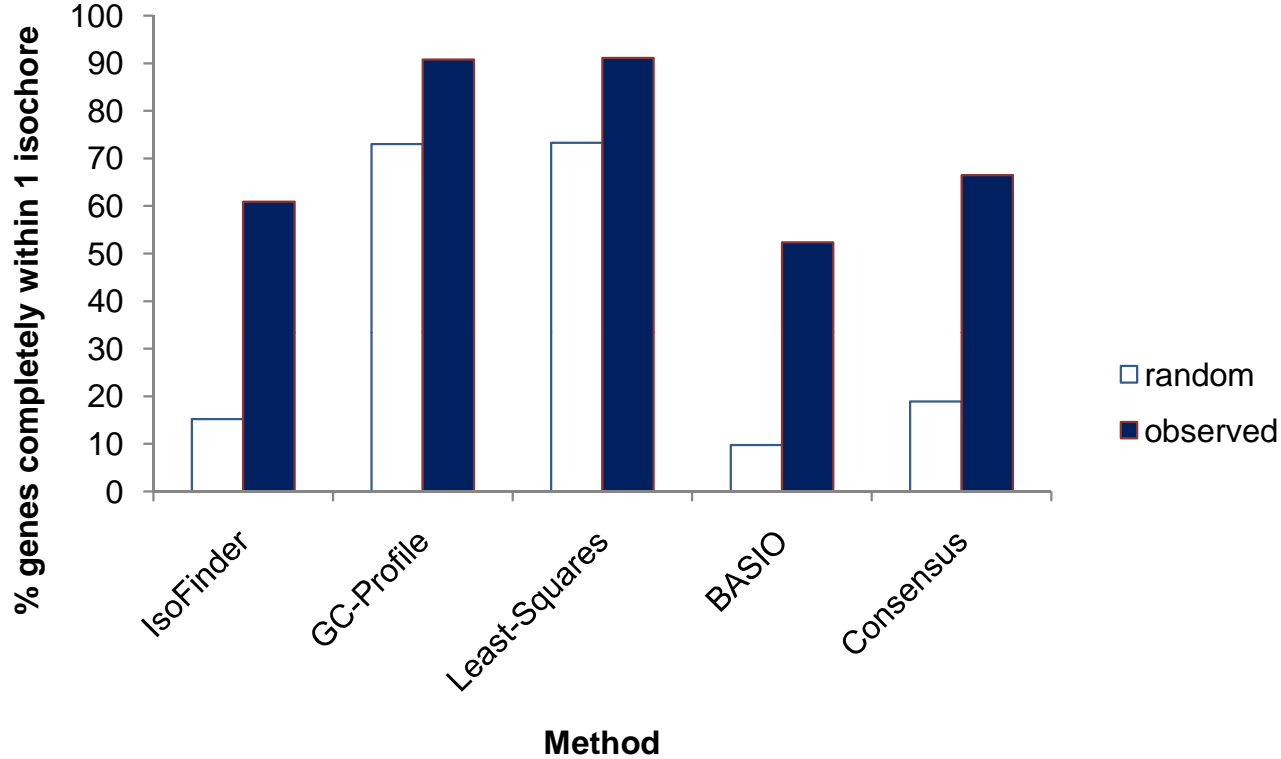

Supplement: Additional data file 2 — For all isochore assignments, more genes reside completely within a single stretch than one would expect by chance. All results are statistically significant (Chi-Square test, all p-values < 0.001). [file gb-2008-9-6-r104-S2.pdf]
