# Supplementary material for: Assignment of isochores for all completely sequenced vertebrate genomes using a consensus
Source: Genome Biol. 2008 Jun 30;9(6):R104. doi: 10.1186/gb-2008-9-6-r104 (PMC2481423; doi:10.1186/gb-2008-9-6-r104)
Supplement: Additional data file 3 — Analysis of isochore families in all genomes and analysis of differences between methods [file gb-2008-9-6-r104-S3.pdf]

Supplementary Material

Content

1. Analysis of isochore families in all genomes

2. Analysis of methods' differences

1. Analysis of isochore families in all genomes

In Figure S 3 we present histograms of the amount of genomic DNA located in segments with a given GC level for the consensus isochore segmentation for all genomes. In the case of warm-blooded vertebrates isochore families (as previously experimentally described) clearly stand out. The observed local maxima obtained from the peaks in the Figures S 3 are in fair agreement with the isochore families reported and used in the field over the last three decades [45-47] throughout all genomes. Possible evolutionary reasons for this conservation are discussed in [28]. Hypotheses about isochore evolution such as the biased gene conversion (BGC), mutation bias or selection, are reviewed in [24].

In the case of fishes (here *D. rerio*, *O. latipes*, *G. Aculeatus*, *T. nigroviridis*), we confirm that cold-blooded vertebrates show a remarkably less heterogeneous composition than warm-blooded ones [46, 48]. In each fish genome analyzed at most two isochore families can be observed. This is in agreement with large-scale experimental analysis of Bucciarelli et al [49]. The genomic differences and hence the preference of different isochore families are assumed to be related to the ecological niche where the respective fishes are living. The fishes analyzed in this study live at distinct water temperatures, the ones that are comfortable at higher temperatures show a clear bias towards GC richer isochore families.

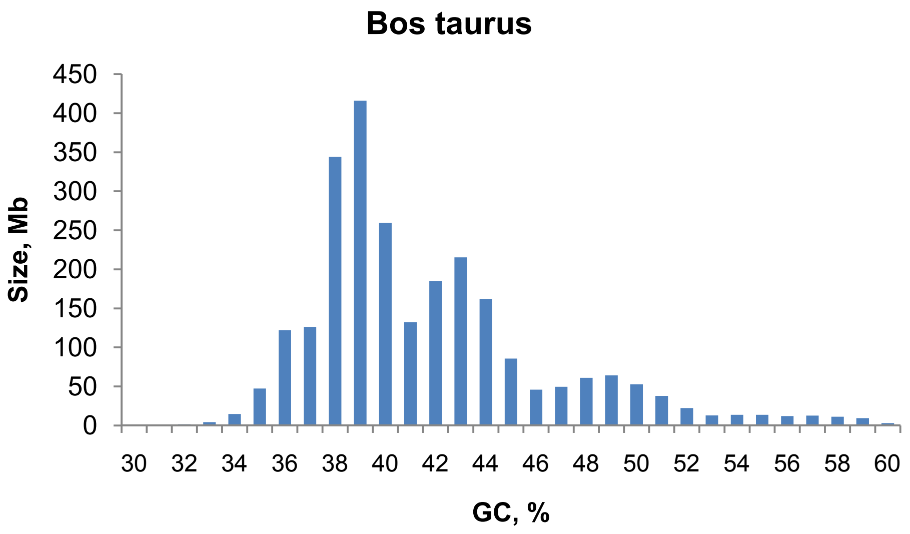
Bos taurus
Size, Mb
450
400
350
300
250
200
150
100
50
0
30 32 34 36 38 40 42 44 46 48 50 52 54 56 58 60
GC, %

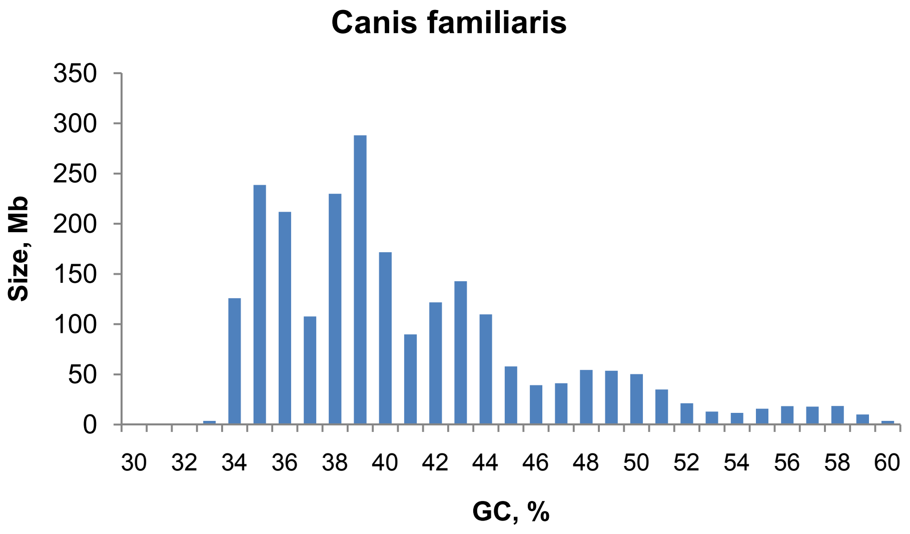
Canis familiaris
Size, Mb
350
300
250
200
150
100
50
0
30 32 34 36 38 40 42 44 46 48 50 52 54 56 58 60
GC, %

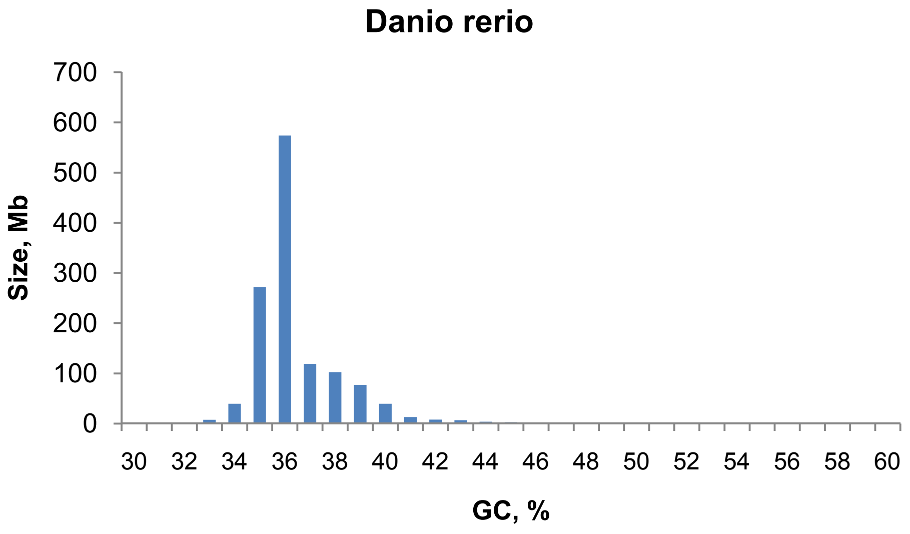
Danio rerio
Size, Mb
700
600
500
400
300
200
100
0
30 32 34 36 38 40 42 44 46 48 50 52 54 56 58 60
GC, %

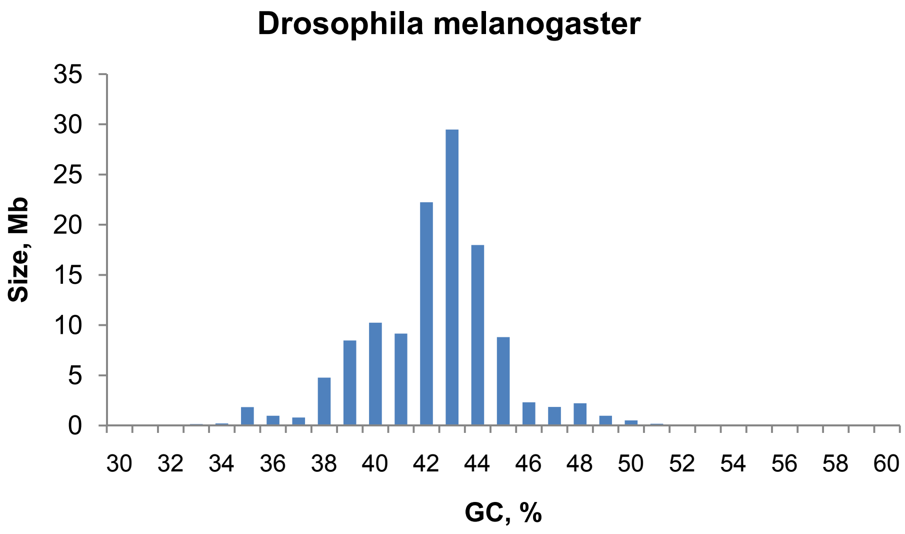
Drosophila melanogaster
Size, Mb
GC, %

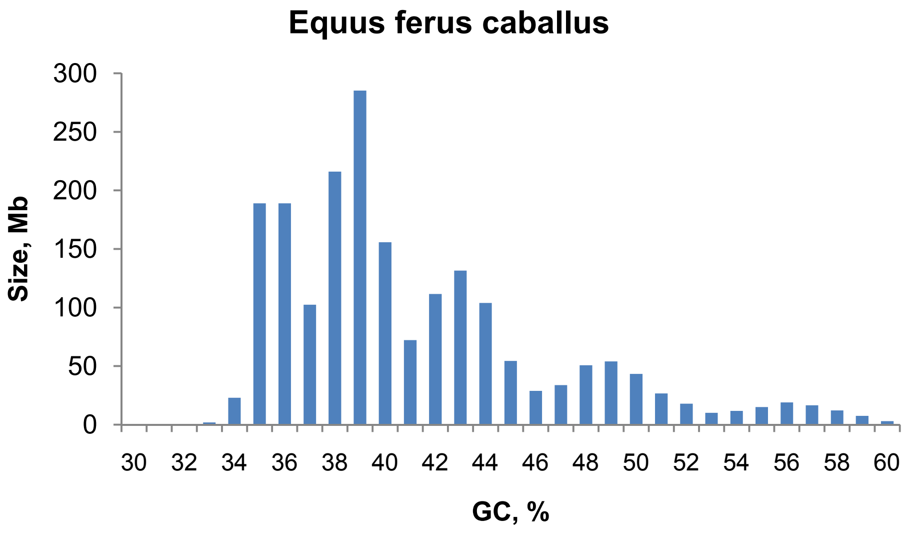
Equus ferus caballus
Size, Mb
GC, %

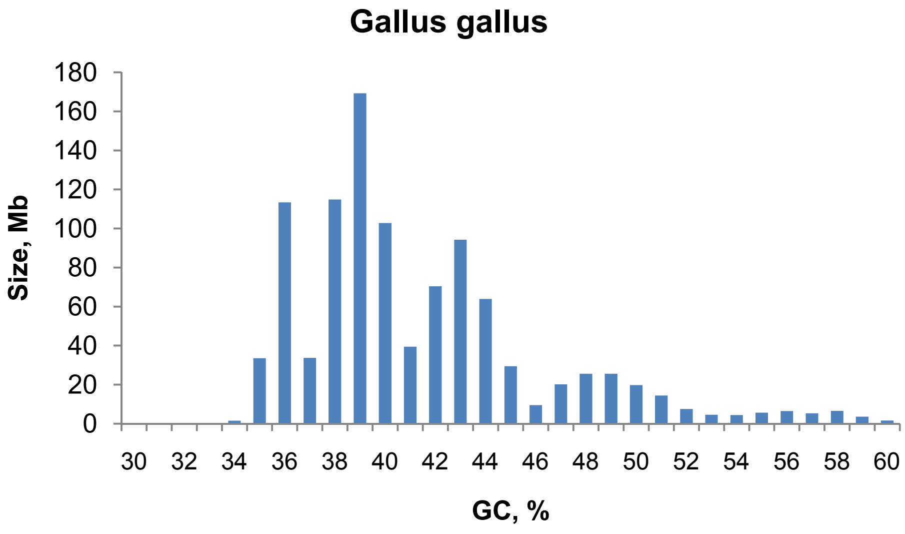
Gallus gallus
Size, Mb
GC, %

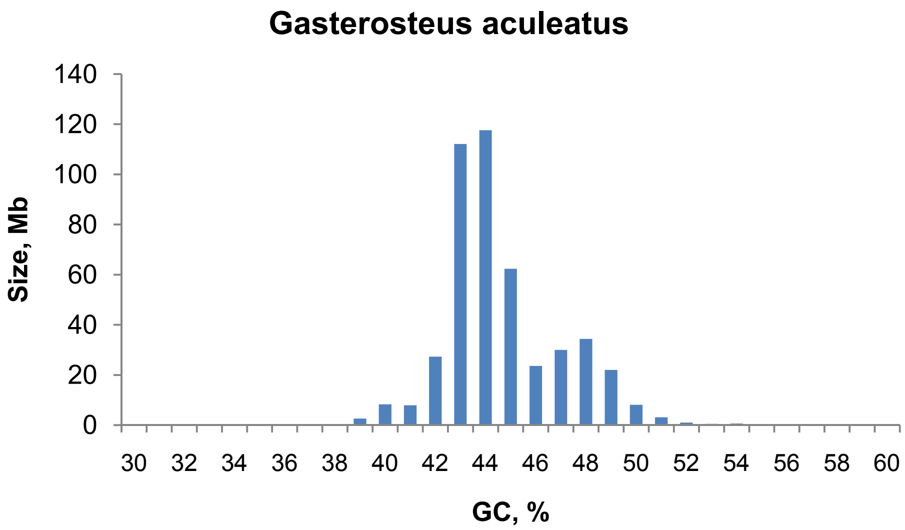
Gasterosteus aculeatus
Size, Mb
GC, %
0
20
40
60
80
100
120
140
30
32
34
36
38
40
42
44
46
48
50
52
54
56
58
60

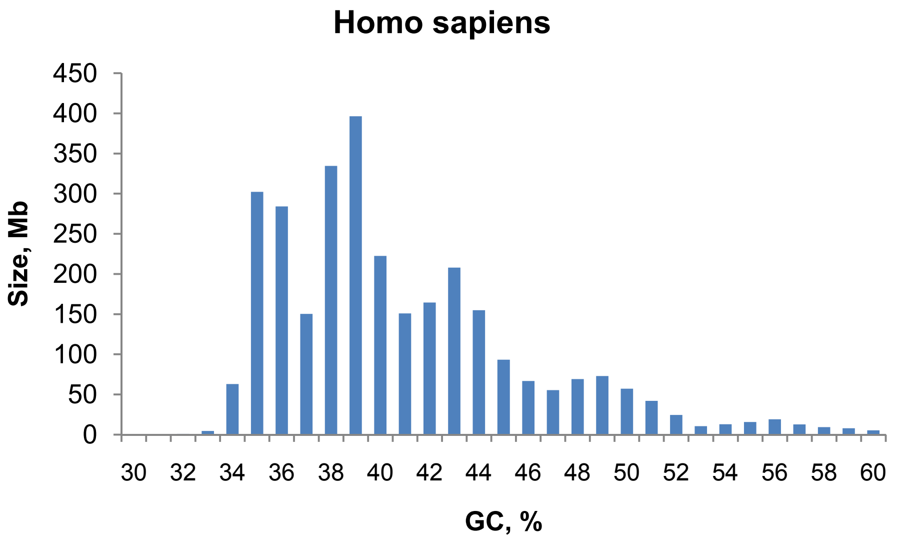
Homo sapiens
Size, Mb
GC, %
0
50
100
150
200
250
300
350
400
450
30
32
34
36
38
40
42
44
46
48
50
52
54
56
58
60

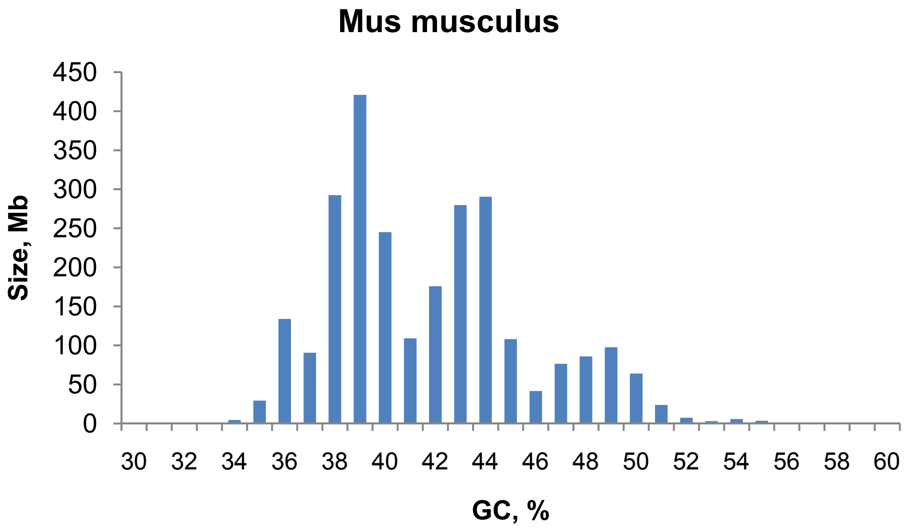
Mus musculus
Size, Mb
GC, %
0
50
100
150
200
250
300
350
400
450
30
32
34
36
38
40
42
44
46
48
50
52
54
56
58
60

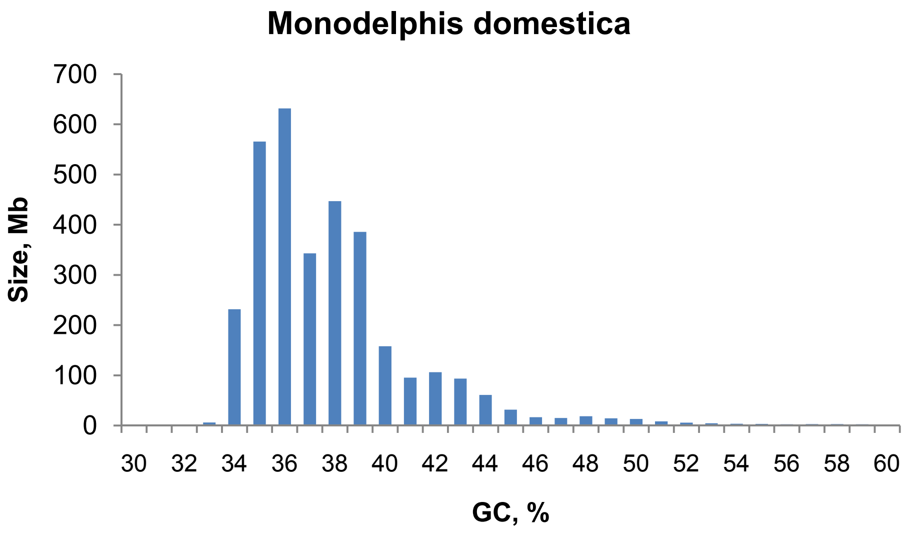
Monodelphis domestica
Size, Mb
700
600
500
400
300
200
100
0
30
32
34
36
38
40
42
44
46
48
50
52
54
56
58
60
GC, %

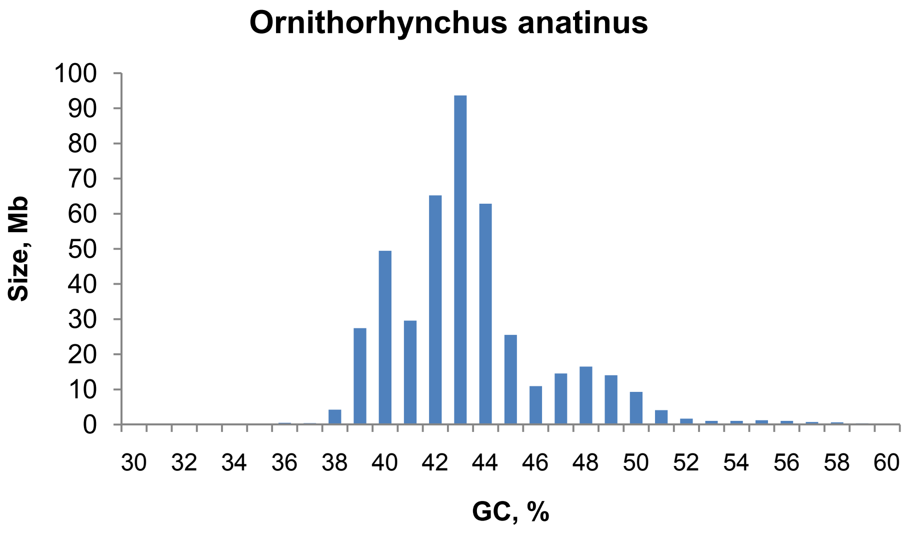
Ornithorhynchus anatinus
Size, Mb
100
90
80
70
60
50
40
30
20
10
0
30
32
34
36
38
40
42
44
46
48
50
52
54
56
58
60
GC, %

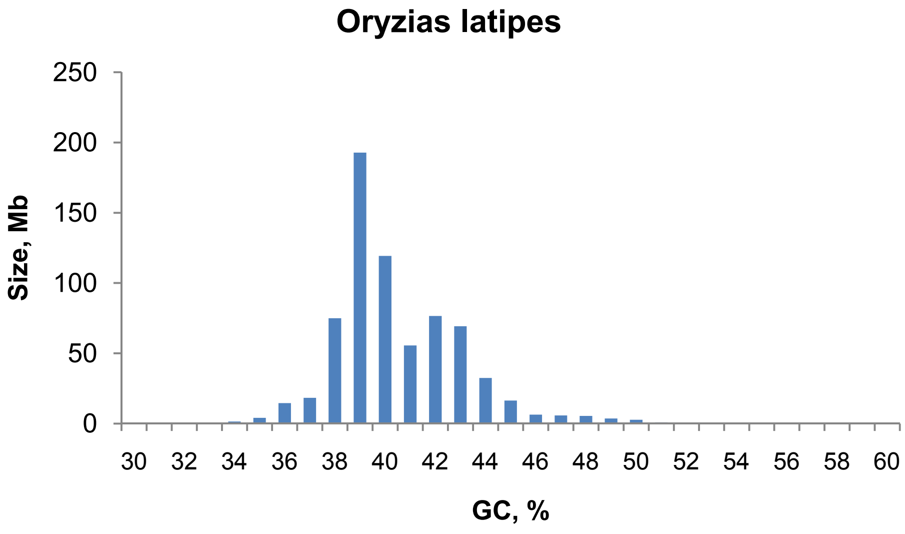
Oryzias latipes
Size, Mb
250
200
150
100
50
0
30
32
34
36
38
40
42
44
46
48
50
52
54
56
58
60
GC, %

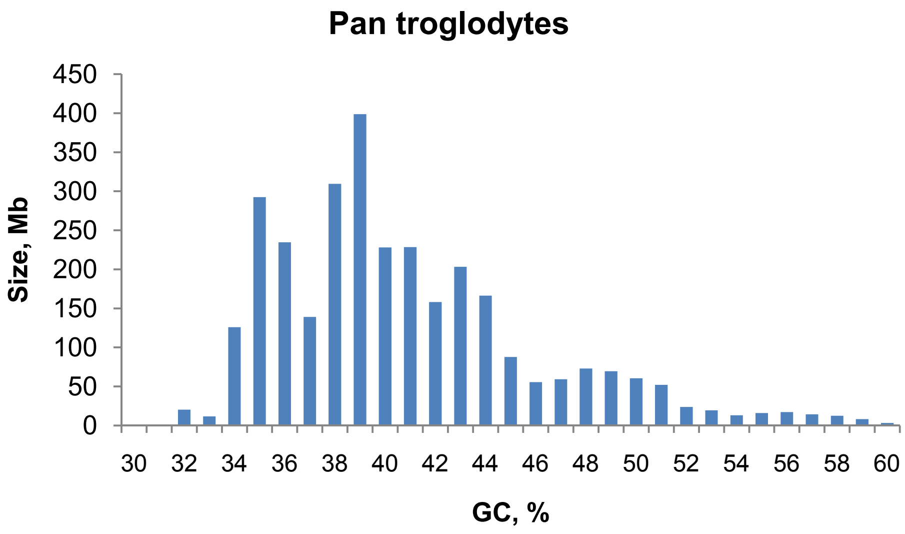
Pan troglodytes
Size, Mb
0
50
100
150
200
250
300
350
400
450
30
32
34
36
38
40
42
44
46
48
50
52
54
56
58
60
GC, %

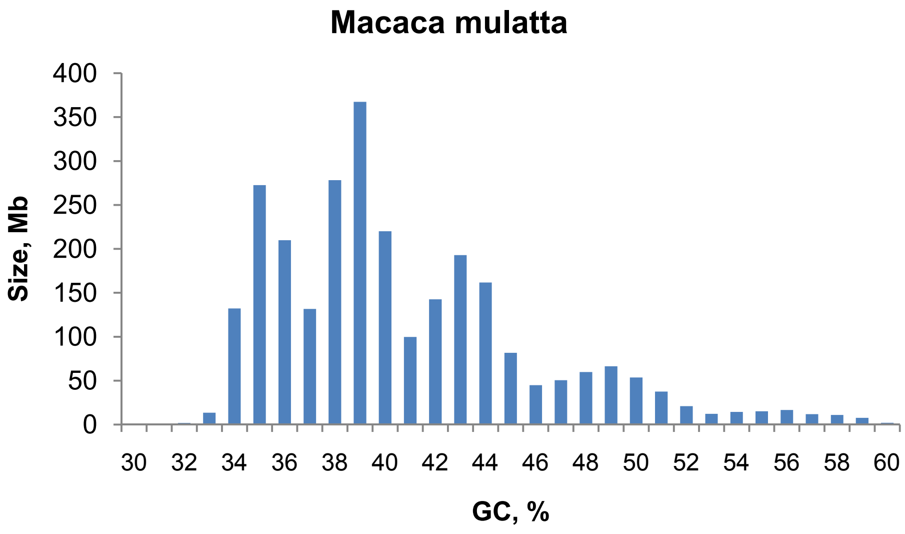
Macaca mulatta
Size, Mb
0
50
100
150
200
250
300
350
400
30
32
34
36
38
40
42
44
46
48
50
52
54
56
58
60
GC, %

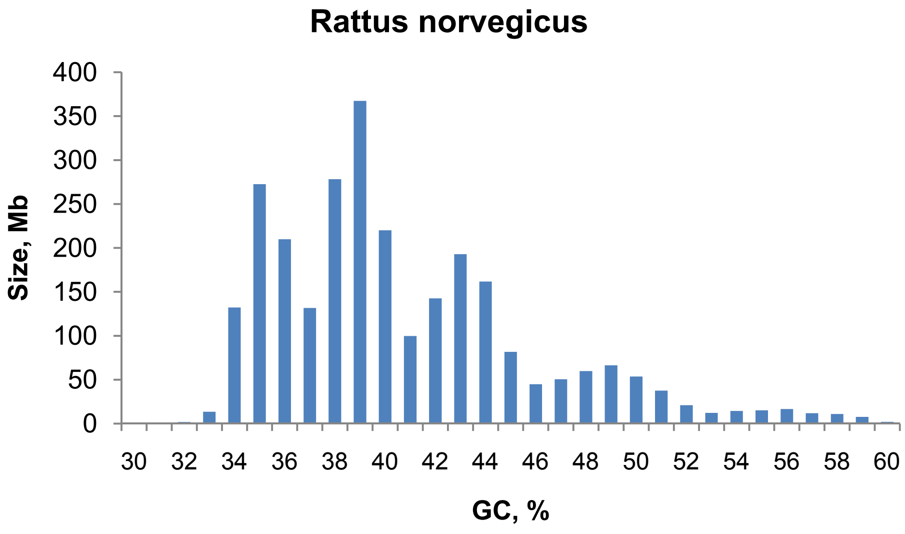
Rattus norvegicus
Size, Mb
0
50
100
150
200
250
300
350
400
30
32
34
36
38
40
42
44
46
48
50
52
54
56
58
60
GC, %

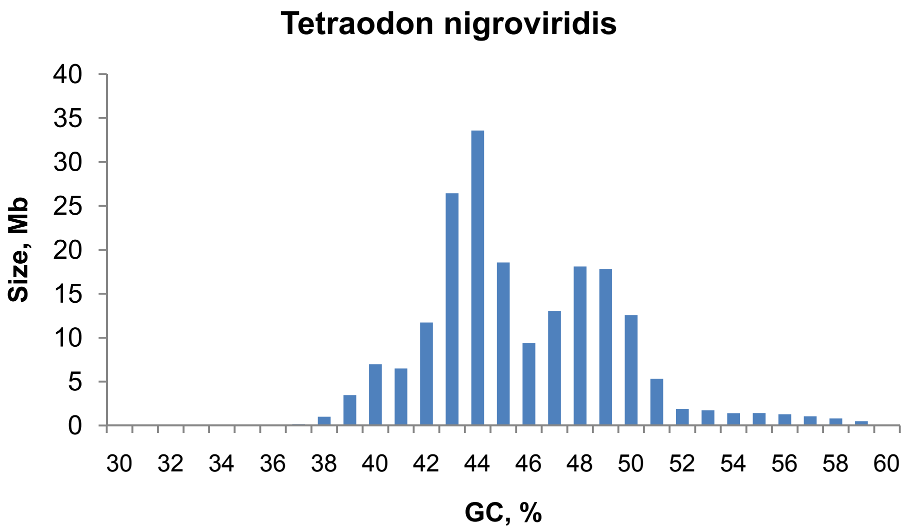

Figure S 3 Distribution of genomic segments according to GC levels in the consensus maps. Genome sizes are calculated from the sum of the segments within the pooled GC bins.

2. Analysis of methods' differences

We evaluated the contribution and the overlap of each method in all genomes to the resulting consensus map. As evaluation measure we used the conditional entropies as proposed by Haiminen et al [44] to compare segmentations. The conditional entropy values indicate the amount of information that a segmentation gives about another one. To be precise, "the conditional entropy of segmentation P given segmentation Q is the expected amount of information we need to identify the segment of P a point belongs to, given that we know the segment of this point in Q"[44]. Figure S 4 shows the conditional entropy values of all methods and genomes. It can be immediately seen, that all four methods are clearly distinct and make a complementary contribution to the consensus map throughout all genomes.

We further evaluated whether the differences of the contributing methods are significantly distinguishable or whether the differences are merely by chance. Indeed, a SimpleLogistics [50] classifier (using the WEKA implementation version 3.5.7 [51] with default parameters) is able to correctly distinguish all four methods with high accuracy of more than 80%. As input features the conditional entropy values were used. The dataset is balanced as all four methods were applied in all genomes. The evaluation was done by ten-fold cross-validation. This shows (with respect to entropy information criteria), that all methods are predominately disjunctive.

Additionally, Table S 1 shows a break-down of the classifier performance for each of the four isochore assignment approaches. It can be seen, that all four methods i.e. IsoFinder, GC-Profile, Least-Squares and BASIO are correctly distinguishable with high precision (78% to 88%). This demonstrates the significant differences and the complementary contribution of the used datasets in the consensus approach.

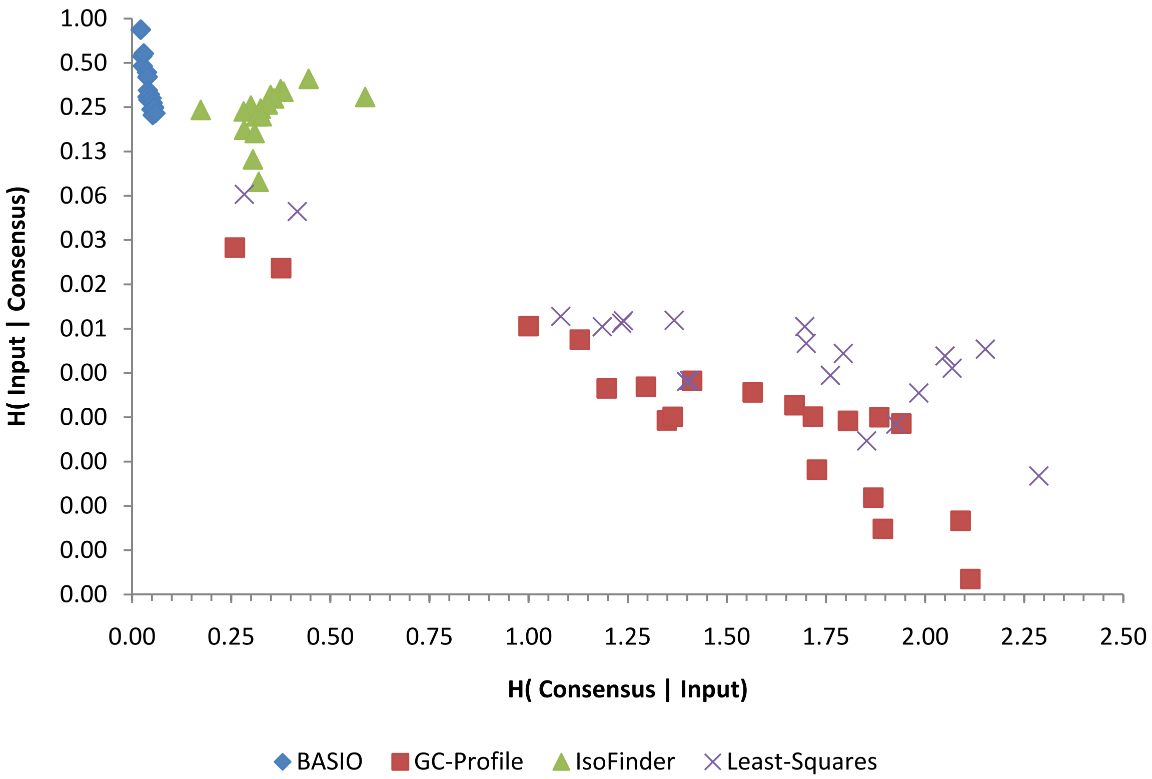

Figure S 4 Conditional entropies of all four isochore maps that were used as input in relation to the consensus map. All four approaches (BASIO, GC-Profile, IsoFinder and Least-Squares) are forming distinct clusters with regard to their entropy values and contribution to the consensus map.

Table S 1 Separation performance of the input approaches

TP-Rate	*FP-Rate*	*Precision*	*Recall*	*F-Measure*	*ROC-Area*	*Class*
90%	9%	78%	90%	84%	97%	BASIO
90%	7%	82%	90%	86%	97%	GC-Profile
71%	5%	80%	71%	75%	96%	IsoFinder
75%	4%	88%	75%	81%	96%	Least-Squares

TP-Rate: true positive rate; FP-Rate: false positive rate; ROC-Area: Receiver Operator Characteristics Area, higher values e.g. >70% indicate excellent classification performance; all measures e.g. Precision, Recall and F-Measure as defined in [51].
